# MRI analysis of tibial PCL attachment in a large population of adult patients: reference data for anatomic PCL reconstruction

**DOI:** 10.1186/s12891-016-1232-3

**Published:** 2016-09-05

**Authors:** Yuanjun Teng, Laiwei Guo, Meng Wu, Tianen Xu, Lianggong Zhao, Jin Jiang, Xiaoyun Sheng, Lihu Xu, Bo Zhang, Ning Ding, Yayi Xia

**Affiliations:** 1 Department of Orthopaedics, the Second Hospital of Lanzhou University, Lanzhou City, No. 82 Cuiyingmen, Chengguan District, Lanzhou City, Gansu Province 730030 China; 2 Orthopaedics Key Laboratory of Gansu Province, the Second Hospital of Lanzhou University, Lanzhou University, Lanzhou City, Gansu Province 730030 China

**Keywords:** Magnetic resonance imaging, Posterior cruciate ligament, PCL reconstruction, Reference data

## Abstract

**Background:**

Consistent reference data used for anatomic posterior cruciate ligament (PCL) reconstruction is not well defined. Quantitative guidelines defining the location of PCL attachment would aid in performing anatomic PCL reconstruction. The purpose was to characterize anatomic parameters of the PCL tibial attachment based on magnetic resonance imaging (MRI) in a large population of adult knees.

**Methods:**

The PCL tibial attachment site was examined in 736 adult knees with an intact PCL using 3.0-T proton density–weighted sagittal MRI. The outcomes measured were the anterior-posterior diameter (APD) of the tibial plateau; angle between the tibial plateau and the posterior tibial ‘shelf’ (the slope where the PCL tibial attachment site was) (PTS); length of the PTS; proximal, central, and distal PCL attachment positions as well as the width of the PCL attachment site; and vertical dimension of the PCL attachment site inferior from the tibial plateau.

**Results:**

The average APD of the tibia plateau was 33.6 ± 3.5 mm, yielding significant differences between males (35.5 ± 3.0 mm) and females (31.6 ± 2.7 mm), *P* <.05, and there was a significantly decreasing trend with increasing age in males (*P* <.05). Mean angle between the tibial plateau and the PTS was 122.4° ± 8.1°, and subgroup analysis showed that the young group had a differently smaller angle (120.9° ± 7.5°) than the middle-aged (123.7° ± 8.2°) and the old (123.4° ± 7.7°) in males population, while there were no significant differences between sexes (*P* >.05). The proximal, central positions and width of the PCL attachment site were 13.4 ± 3.0 mm, 17.8 ± 3.0 mm and 9.6 ± 2.4 mm along the PTS, with significant differences between males and females (*P* <.05), and accounted for 60.0 % ± 9.1 %, 80.0 % ± 4.6 % and 43.3 % ± 9.7 % of the PTS respectively, with no significant differences between sexes and among age groups (all *P* >.05).

**Conclusions:**

This study provides reference data of the tibial PCL attachment based on MRI in the sagittal orientation. In analysis of retrospective data from a large population of adult patients, the quantitative values can be used as references to define the inserted angle and depth of the drill guide, and the exact position and size of the tibial PCL tunnel for performing arthroscopic anatomic PCL reconstruction.

## Background

The posterior cruciate ligament (PCL) is composed of two bundles (the anterolateral bundle and the posteromedial bundle), and is regarded as the central pivot point of the knee and acts as an essential role in joint stability of knee [1]. Biomechanical studies have indicated that the PCL contributes 95 % restraint to posterior translation of tibia [2]. The PCL injury is not as common as anterior cruciate ligament (ACL) injury in high-energy knee trauma. It has been reported that the incidence of PCL injury varies from 1 to 44 % in all knee ligament injuries [2, 3]. And PCL injury presents more frequently in trauma population (as high as 37 %) than that in athletic injury population [4, 5].

The surgical management arthroscopically has been increasingly recommended for PCL tears. Compared with the nonoperative treatment, PCL reconstruction is the preferred treatment for patients with severe or complex PCL injuries [2, 6]. The possible indications for PCL reconstruction include: an acute PCL tear in conjunction with a knee dislocation, or with an anteroposterior laxity ≥12 mm on the PCL stress radiograph, a complete PCL tear with a stress radiographic anteroposterior laxity ≥8 mm and combined with repairable meniscal tear, and a chronic PCL tear with a stress radiographic anteroposterior laxity ≥8 mm with functional limitations [7–13]. Additionally, anatomic PCL reconstruction, which needs to make the tunnels exactly at the attachments of the native PCL, has obtained better clinical outcomes than the nonanatomic reconstruction [14–16]. Currently, two widely used techniques are used to perform anatomic PCL reconstruction: the single-bundle PCL reconstruction and the double-bundle PCL reconstruction. Though several level-2 and −3 studies have quantified potential differences between the two techniques, their conclusions were controversial [8, 17–22].

Regardless of the single-bundle or double-bundle technique, sufficient knowledge of normal PCL anatomy is essential to ensure correct tunnel placement during anatomic PCL reconstruction. Several small sample cadaveric studies have investigated the anatomic characteristics of the anterolateral and posteromedial bundles of the PCL at the femoral attachments [7, 8, 23, 24]. However, few studies have described relevant data of the PCL at the tibial attachment. Therefore, the purpose of this study was to characterize anatomic parameters of the PCL tibial attachment based on MRI in a large population of adult knees. We hypothesized that it would provide some useful reference data to assist orthopaedic surgeons with intraoperative and postoperative assessments of correct tunnel placement during arthroscopic anatomic PCL reconstruction.

## Methods

### Patient selection

Ethics approval for this study was granted by the institutional review board of our hospital. The inclusion criteria were: (1). patients performed 3.0 Tesla knee MRI; (2). Time between June 2010 and April 2015; (3). patients’ age was not less than 18. We excluded the patients with skeletal dysplasia, a displaced fracture around knee, previous knee surgery, imaging evidence of degenerative joint disease, or knee abnormalities caused by any disease.

### Magnetic resonance imaging

All the included patients were performed by 3.0 Tesla MRI according to the standard clinical knee MRI protocols (Siemens AG, Munich). All patients were scanned with coronal, sagittal and axial MR images (matrix: 512 × 512 or 256 × 256, FOV: maximum to 200 mm, slice thickness: 2.5–4 cm) /T1-weighted images (TR/TE = 400–750 msec / 17–30 msec), T2-weighted images (TR/TE = 1800–3500 msec / 60–100 msec) and a turbo spin-echo pulse sequence was used to obtain proton density-weighted images (TR/TE = 1800–3500 msec / 19–40 msec).

### Quantitative anatomic measurements

**Fig. 1:**
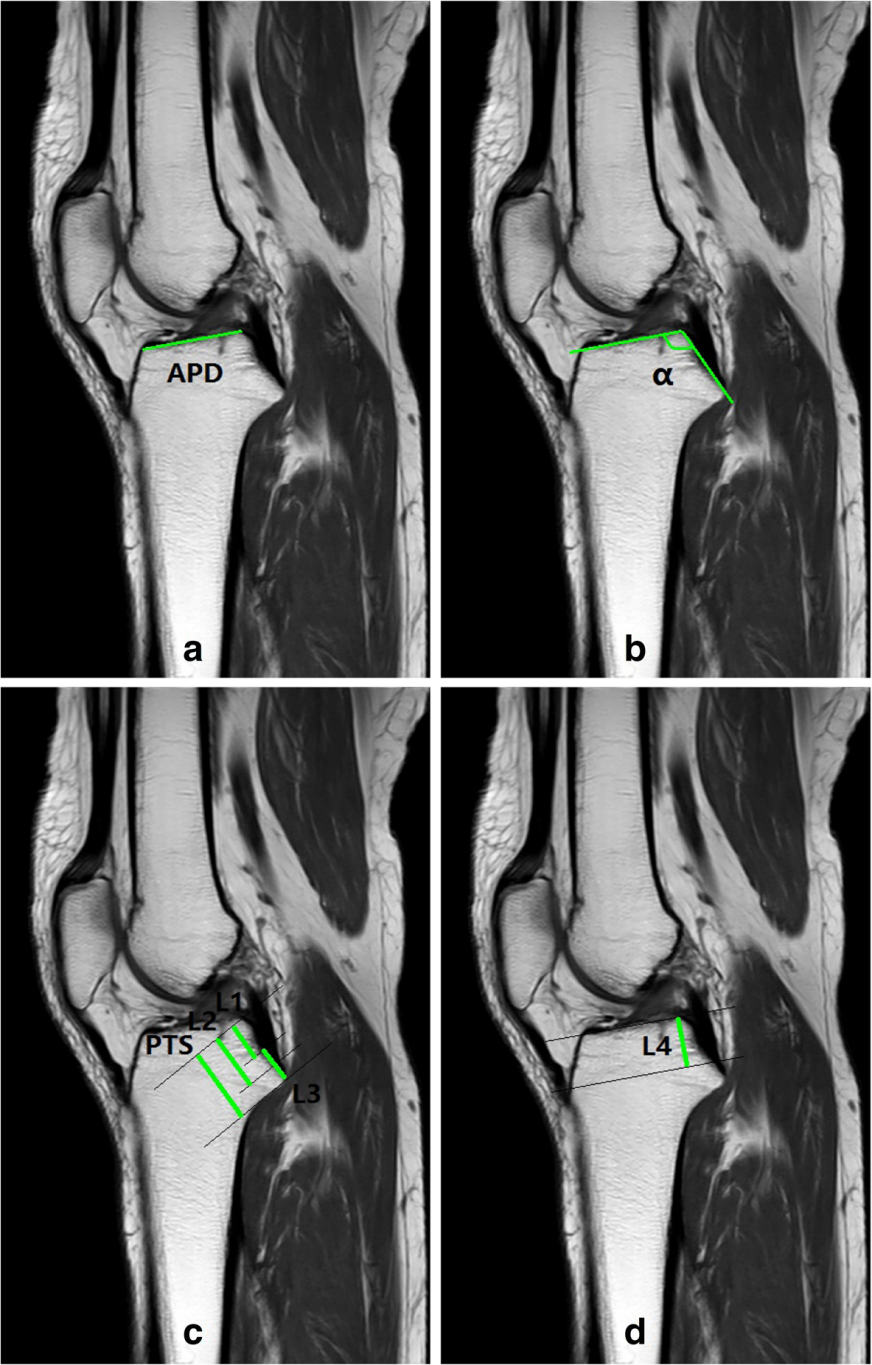
The relevant measurements of the PCL tibial attachment. **a** MRI showing measurement of the APD of the tibial plateau; **b** MRI showing measurement of the angle (α) between the tibial plateau and PTS (posterior tibial ‘shelf’); **c** MRI showing measurement of the distance from the proximal (L1) and central (L2) positions of the tibial PCL attachment to the tibial plateau, the width (L3) of the attachment, and the length of the PTS; **d** MRI showing measurement of the vertical dimension (L4) of the PCL central attachment site from the tibial plateau

**Fig. 2:**
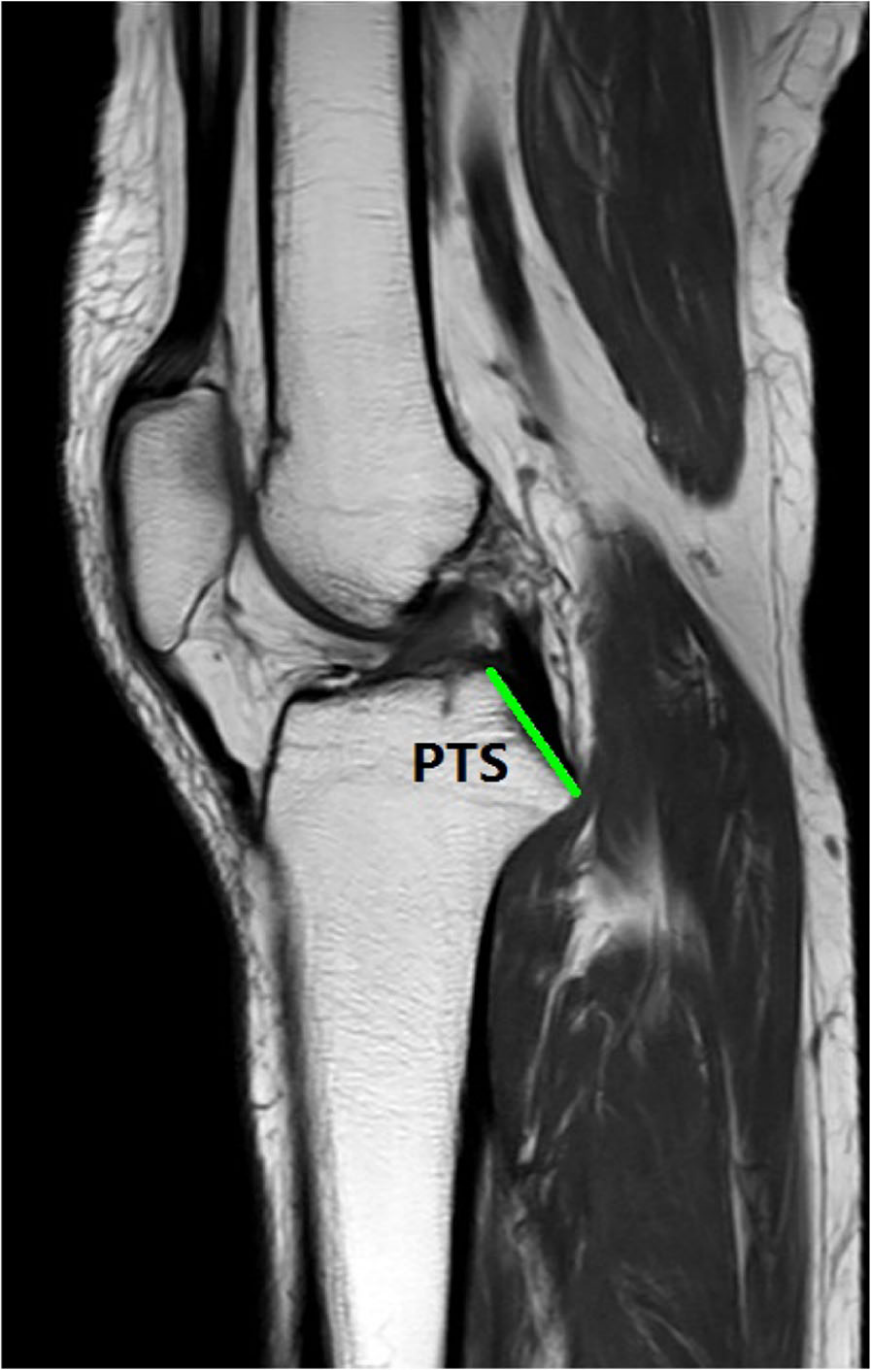
PTS (posterior tibial ‘shelf’): the slope where the PCL tibial attachment site was

Quantitative anatomic measurements were performed digitally with RadiAnt DICOM Viewer (Medixant, Poznan, Poland) software. Measurements were taken on the T1-weighted sagittal MRI slice that provided the most inclusive view of the PCL tibial attachment. The relevant measurements of the PCL tibial attachment were performed (Fig. 1) referring to an earlier study focus on the measurements of anterior cruciate ligament (ACL) tibial attachment, by Frank et al. [25], including the APD of the tibial plateau, the angle between the tibial plateau and the posterior tibial ‘shelf’ (the slope where the PCL tibial attachment site was) (PTS) [26] (Fig. 2), the length of the PTS. The other data measured were the proximal, central and distal positions of the PCL tibial attachment, the width of the attachment site, and the vertical dimension of the PCL attachment site from the tibial plateau. The APD was measured from the anterior edge to the posterior edge of the tibia plateau. Similarly, the length of the PTS was from the proximal edge to the distal edge of the PTS. The angle was measured between two lines separately along the tibial plateau and the PTS. The proximal, central and distal positions as well as the dimater of the PCL tibial attachment were expressed as a value (mm) along the PTS as well as a percentage of the length of the PTS. As the distal border of the PCL attachment and the distal edge of the PTS was almost the same position on MR images, the same value was defined to the length of the PTS and the distance from the distal position of the attachment to the proximal edge of the PTS. Additionally, the vertical dimension of the PCL attachment site was measured inferior from the center of the PCL attachment perpendicularly to a line along the tibial plateau. Finally, all the measurements were analyzed for the cohort as a whole, as well as the comparison between males and females.

### Reliability analysis

To determine the interobserver reliability of the anatomic measurements, a single author (author 1) measured all parameters of the entire data set (*n* = 736), a second author (author 2) separately performed the measurements of 100 randomly selected cases from the entire data set, blinded to the values from author 1. For the intraobserver reliability, author 1 performed measurements of 40 randomly selected cases from the original data set on a second occasion 4 weeks apart. Throughout the reading session, clinical and demographic information as well as recorded measurements were blinded to the authors.

### Statistical analysis

The data were tabulated on Microsoft Excel (v. 2007; Microsoft Corp, Redmond, WA, USA) and all statistical analyses were performed using SPSS software (v. 19; SPSS Inc, Chicago, IL, USA). As the continuous variables showed approximately normal distributions confirmed from review of frequency tables, results were described by mean ± standard deviation. Independent t test was used when anatomic parameters were compared between sex cohorts, while one-way ANOVA among age cohorts, with a significance level of *P* = .05. Two-way mixed effects model, single-measures intraclass correlation coefficients (ICCs) were calculated to assess inter- and intraobserver reliability.

## Results

Totally, patients with 736 knees were included. Of which, 379 were males and 357 were females. The average age at the time of MRI was 39 ± 13.7 years (range, 18–75 years; males, 18–75 years; females, 18–73 years). There were 389 right knees and 347 left knees.

The detailed anatomic parameters of the PCL tibial attachment between males and females are reported in Table 1. The APD of the tibia plateau, absolute values of proximal, central positions and width of the PCL attachment site along the PTS, as well as PTS itself (also the distal position of the PCL tibial attachment), and mean vertical dimension of the PCL attachment site yielded significant differences between males and females, *P* <.05. The proximal, central positions and width of the PCL attachment when expressed as a percentage of the PTS and mean angle between the tibial plateau and the PTS showed no significant differences between sex cohorts, *P* >.05.

**Table 1:** Anatomic parameters of the PCL tibial attachment compared between males and females^a^

Variable	Mean ± Standard Deviation			*P*
	Total	Male	Female	
APD, mm	33.6 ± 3.5	35.5 ± 3.0	31.6 ± 2.7	.000
α, °	122.4 ± 8.1	122.6 ± 7.9	122.2 ± 8.3	.462
PTS, mm	22.3 ± 3.5	23.6 ± 3.5	20.9 ± 2.8	.000
L1, mm	13.4 ± 3.0	14.2 ± 3.1	12.5 ± 2.6	.000
L2, mm	17.8 ± 3.0	18.9 ± 3.1	16.7 ± 2.5	.000
L3, mm	9.6 ± 2.4	10.1 ± 2.5	9.1 ± 2.1	.000
L1/PTS, %	60.0 ± 9.1	60.2 ± 9.5	59.8 ± 8.8	.513
L2/PTS, %	80.0 ± 4.6	80.1 ± 4.7	79.9 ± 4.4	.513
L3/PTS, %	43.3 ± 9.7	43.0 ± 9.9	43.6 ± 9.4	.383
L4, mm	15.0 ± 3.3	15.9 ± 3.4	14.1 ± 2.8	.000

^a^ *APD* anterior-posterior diameter, *α* the angle between the tibial plateau and the posterior tibial ‘shelf’, *PTS* posterior tibial ‘shelf’, *L1* distance from the proximal position of the tibial PCL attachment to the tibial plateau, *L2* distance from the central position of the tibial PCL attachment to the tibial plateau, *L3* width of the tibial PCL attachment site, *L4* the vertical dimension of the PCL attachment site from the center of the PCL attachment perpendicularly to a line along the tibial plateau

Measurements of the PCL’s tibial attachment in males, stratified into three age groups: the young (age, 18–30 years), the middle-aged (age, 31–50 years) and the old (age, 51–75 years) (Table 2). Mean APD of the tibal plateau, the angle between the tibial plateau and the PTS, the central position and width of the PCL attachment site along the PTS, as well as PTS itself, and the mean vertical dimension of the PCL attachment site showed significant differences between age groups, *P* <.05. While the proximal position of the PCL tibial attachment and percentage forms of the proximal, central positions and width of the PCL tibial attachment showed no significant differences among the three age groups, *P* >.05.

**Table 2:** Anatomic parameters of the PCL tibial attachment among the three age groups in males^a^

Variable	Mean ± Standard Deviation			*P*			
	The Young (*n* = 145)	The Middle-aged (*n* = 170)	The Old (*n* = 64)	Total	The Young vs the Middle-aged	The Young vs the Old	The Middle-aged vs the Old
APD, mm	36.2 ± 3.0	35.4 ± 3.0	34.3 ± 2.9	.000	.021	.000	.010
α, °	120.9 ± 7.5	123.7 ± 8.2	123.4 ± 7.7	.005	.002	.035	.796
PTS, mm	24.4 ± 3.7	23.2 ± 3.5	22.8 ± 3.1	.003	.005	.004	.407
L1, mm	14.6 ± 3.2	14.0 ± 3.1	13.9 ± 3.2	.144	.072	.145	.920
L2, mm	19.5 ± 3.2	18.6 ± 3.0	18.4 ± 2.9	.014	.012	.016	.600
L3, mm	10.6 ± 2.4	10.0 ± 2.6	9.3 ± 2.3	.002	.027	.001	.077
L1/PTS, %	60.0 ± 9.0	60.2 ± 9.8	60.8 ± 9.6	.836	.822	.550	.661
L2/PTS, %	80.0 ± 4.5	80.1 ± 4.9	80.4 ± 4.8	.836	.822	.550	.661
L3/PTS, %	43.8 ± 9.1	43.1 ± 10.5	41.1 ± 10.0	.203	.532	.075	.179
L4, mm	16.7 ± 3.5	15.4 ± 3.3	15.3 ± 3.3	.002	.001	.007	.801

^a^ *APD* anterior-posterior diameter, *α* the angle between the tibial plateau and the posterior tibial ‘shelf’, *PTS* posterior tibial ‘shelf’, *L1* distance from the proximal position of the tibial PCL attachment to the tibial plateau, *L2* distance from the central position of the tibial PCL attachment to the tibial plateau, *L3* width of the tibial PCL attachment site, *L4* the vertical dimension of the PCL attachment site from the center of the PCL attachment perpendicularly to a line along the tibial plateau

Data comparing anatomic parameters of the tibial PCL attachment among the three age groups in females are shown in Table 3. However, no significant differences were found in the three age groups, all *P* >.05.

**Table 3:** Anatomic parameters of the PCL tibial attachment among the three age groups in females^a^

Variable	Mean ± Standard Deviation			*P*
	The Young (*n* = 91)	The Middle-aged (*n* = 171)	The Old (*n* = 95)	Total
APD, mm	32.0 ± 2.5	31.4 ± 2.6	31.6 ± 2.9	.143
α, °	120.8 ± 8.4	122.2 ± 7.7	123.4 ± 9.1	.114
PTS, mm	21.0 ± 2.6	20.7 ± 2.9	21.2 ± 2.9	.383
L1, mm	12.4 ± 2.3	12.4 ± 2.6	12.8 ± 2.8	.387
L2, mm	16.7 ± 2.2	16.6 ± 2.5	17.0 ± 2.7	.359
L3, mm	9.2 ± 2.2	9.1 ± 2.1	9.0 ± 2.1	.779
L1/PTS, %	59.3 ± 9.2	59.8 ± 8.8	60.4 ± 8.3	.719
L2/PTS, %	79.7 ± 4.6	79.9 ± 4.4	80.2 ± 4.1	.719
L3/PTS, %	44.0 ± 9.6	43.9 ± 9.3	42.9 ± 9.6	.674
L4, mm	14.2 ± 2.4	13.9 ± 2.8	14.1 ± 3.1	.727

^a^ *APD* anterior-posterior diameter, *α* the angle between the tibial plateau and the posterior tibial ‘shelf’, *PTS* posterior tibial ‘shelf’, *L1* distance from the proximal position of the tibial PCL attachment to the tibial plateau, *L2* distance from the central position of the tibial PCL attachment to the tibial plateau, *L3* width of the tibial PCL attachment site, *L4* the vertical dimension of the PCL attachment site from the center of the PCL attachment perpendicularly to a line along the tibial plateau

Inter- and intraobserver reliability of tibial anatomic parameters based on ICCs was shown in Table 4. It was assessed to be of high inter- and intraobserver reliability for each measurement with interobserver ICCs ranged from 0.845 to 0.934 and intraobserver ICCs from 0.825 to 0.927.

**Table 4:** Inter- and intraobserver reliability of tibial anatomic parameters based on ICCs^a^

	ICC	
	Interobserver	Intraobserver
APD	0.910	0.924
α	0.845	0.896
PTS	0.906	0.927
L1	0.934	0.921
L2	0.892	0.873
L3	0.886	0.893
L4	0.907	0.825

^a^ *ICCs* intraclass correlation coefficients, *APD* anterior-posterior diameter, *α* the angle between the tibial plateau and the posterior tibial ‘shelf’, *PTS* posterior tibial ‘shelf’, *L1* distance from the proximal position of the tibial PCL attachment to the tibial plateau, *L2* distance from the central position of the tibial PCL attachment to the tibial plateau, *L3* width of the tibial PCL attachment site, *L4* the vertical dimension of the PCL attachment site from the center of the PCL attachment perpendicularly to a line along the tibial plateau

## Discussion

The most important finding of this study was the measurements of the APD and the tibial angle, as well as the determination of normal position of the tibial PCL attachment relative to the sagittal depth of the PTS based on MRI in a large population of adult knees. MRI have proven to be a very accurate technique for diagnosing PCL tears, especially for acute tears [27], with sensitivity values of 100 % and specificity values of 97 to 100 % [28–32]. With thorough MRI information of the PCL attachment, it would provide some reference data to assist orthopaedic surgeons with intraoperative and postoperative assessments of correct tunnel placement during arthroscopic anatomic PCL reconstruction.

On the sagittal plane of the MRI, our study showed that the APD of the tibial plateau was measured at ~34 mm, a little greater in males (~36 mm) than that in females (~32 mm). When stratified into three age groups, the value of the APD showed a significantly decreasing trend with age in males. The author doubts whether it was due to the degeneration changes of the cartilage as each individual goes through the aging process. It needs further research to clarify the issue. One recent study by Osti et al. [33] showed that the anteroposterior diameter of the tibial plateau was 57.43 ± 3.69 mm on radiographs with 20 nonpaired, fresh-frozen human cadaveric knee specimens, while Lorenz et al. [14] measured the anteroposterior diameter of the tibial plateau in true lateral radiography to be 61 mm based on 16 human cadaver specimens. The two studies both performed the largest anteroposterior diameter of the tibial plateau from the anterior edge of the tibia to the posterior tibial cortex. In another study by Frank et al. [25], the anteroposterior diameter of the tibia was 50 ± 4 mm, measured from the anterior edge of the tibia at the articular margin, proximal to the tibial tubercle, to the tibial origin of PCL. Differences with values of these studies were resulted from the fact that we used a different APD measurement technique in the present study. We measured the APD of the tibial plateau from the anterior edge to the posterior edge of the tibia plateau (as shown in Fig. 1). With this value, we can decide the insertion depth of the drill guide along the tibial pateau to reach the tibial PCL attachment, so it would be helpful to find the attachment of the PCL during arthroscopic PCL reconstruction.

The angle between the tibial plateau and the PTS was ~122°, with no significant differences between males and females. However, the young group had a differently smaller angle than the middle-aged and the old group. The explanation might be the appearance of slight knee hyperplasia in the middle-aged and old group. The measurements of the APD and the angle described above might be useful for defining the angle and depth of the drill guide during arthroscopic PCL reconstruction.

On the surface of the posterior tibial ‘shelf’, we performed measurements of the distance from the proximal, central as well as distal positions (also the length of the PTS) of the tibial PCL insertion to the tibial plateau and the width of the PCL attachment. The PCL attachment was measured to be 9.6 ± 2.4 mm on the MR images in our study, which is consistent with the findings from Osti et al. [33] by cadaveric knee specimens. They found that the superoinferior value of the PCL attachment was 9.58 ± 1.60 mm. Additionally, we found that there were statistically significant differences in all sites examined between males and females when the results were expressed in absolute values. However, no significant differences were found between the two groups when using the percentage of total depth of the PTS. It is likely due to differences of tibia size between males and females. The smaller size of tibia in females resulted in statistical differences when compared with males in absolute numbers. When the measurements were expressed as a percentage of tibial size, the outcomes were then standardized and the differences no longer presented. The results were similar with those from Frank et al. [25], who found statistically significant differences in tibial ACL attachment positions between males and females when the measurements were expressed in absolute values, and noted no differences when expressed as percentage of tibial APD. Therefore, a percentage measurement which allows for standardization of the positions, regardless of patient sex or size, might be more clinically significant and practical.

When stratified into three age groups in males, the values of the central, distal positions (also the length of the PTS) and width of the tibial PCL insertion along the PTS were greater in the young group than the middle-aged and the old. However, the value of the proximal position of the tibial PCL attachment showed no significant differences among age groups. Additionally, based on the 736 MR images, the values of the proximal, central positions and the width of the tibial PCL attachment accounted for 60.0 ± 9.1, 80.0 ± 4.6 and 43.3 ± 9.7 % of the PTS, respectively, regardless of sex or age. It means that the center of tibial PCL attachment locates ~ 80 % along the PTS distal from the posterior edge of the tibial plateau and covers ~43 % of the PTS. Therefore, for tibial tunnel placement when performing anatomic PCL reconstruction, we should strive for an accurate position centered at the midpoint of the normal tibial PCL attachment (~80 %), and pay more attention not to extending outside of this range (~43 %).

However, absolute values of the PCL attachment on the PTS might be more practical during arthroscopic PCL reconstruction. Based on our results in this study, when position the PCL attachment on the PTS, the drill guide should insert to a depth of ~18.9 mm in males, while ~16.7 mm in females. And the diameter of the tibial PCL tunnel should not be more than 10.1 and 9.1 mm respectively in males and females. These might be the most useful information for intraoperative and postoperative assessments of correct tunnel placement during arthroscopic anatomic PCL reconstruction. However, whether these data are clinically accurate and will lead to good clinical outcomes, more studies are still needed to validate the efficacy of our work.

Additionally, we performed the measurement of the width of PCL attachment in the sagittal orientation. Combining with the mediolateral value of the PCL attachment from Osti et al. [33], maybe we could determine the approximate position and size of tibial tunnel for PCL reconstruction. However, as the size and shape of the PCL attachment site are different from patient to patient [24, 34–36], and different shapes of the PCL attachment site may require different types of reconstruction to ensure proper attachment restoration, we should define the accurate outline of the PCL attachment site and perform the measurements in multiple orientations in future research. This can help to choose proper type and size of the graft and perform more individual and accurate PCL reconstruction [37].

The vertical dimension from the PCL attachment center to the tibial plateau was 15.0 ± 3.3 mm in this study. We did not search the similar measuring method and results about this parameter. However, several relevant studies about this parameter can still provide useful information for PCL reconstruction. Lorenz et al. [14] performed the perpendicular distance of the geometric insertion point to the tangent of the medial tibial plateau on lateral X-ray images, and the value was 8 mm on average, almost twice the size compared with the result in the present study. The differences might be due to the inclusion of tibial intercondylar eminence and the appearance of cartilage and soft tissue on MR images. In a quantitative analysis of the anterolateral and posteromedial bundles’ insertion areas, Johannsen et al. [23] measured the vertical dimension from the tibial PCL attachment center to a line intersecting the champagne glass drop-off ridge [38] perpendicular to the tibial axis line, and the value was 5.5 ± 1.7 mm. However, they did not report the vertical distance from the PCL center site to the tibial plateau. As a supplement to the results by Johannsen et al. [23], the present study provided more reference data for PCL reconstruction.

### Limitations and future directions

We acknowledge that this study has some limitations. First, since it was a retrospective study, the weight and height of the patient could not be acquired, which prevented us from comparing males and females in equal size. Second, all our measurements were performed on the sagittal slice of the MRI, which cannot fully represent anatomy of the PCL tibial attachment as 3-dimensional reconstruction. However, routine preoperative 2-dimensional MRI is commonly performed to identify knee injury as 3-dimensional MRI of the knee is not feasible at most centers. Therefore, we performed the measurements of the reference parameters on 2-dimensional MRI. Third, the present study merely used MRI to determine the position of the tibial PCL attachment without relevant anatomic data. Therefore, there might be some small deviations between the estimated position of the tibial PCL attachment and the actual insertion. However, the degree of deviation would be limited for the current sensitivity and specificity of MRI, and the reliability analysis we conducted in the present study made the results more reliable. In addition, the patients included were from a single ethnicity-Chinese Han nationality, therefore, there might be some limitations when the reference data were used for other ethnicities. In future research, we should include patients from multiple ethnicities and record the weight and height of the patients, so that we could collect more comprehensive information for research when needed.

## Conclusion

This study provides reference data of the tibial PCL attachment based on MRI in the sagittal orientation. In analysis of retrospective data from a large population of adult patients, the quantitative values can be used as references to define the inserted angle and depth of the drill guide, and the exact position and size of the tibial PCL tunnel for performing arthroscopic anatomic PCL reconstruction.

## Abbreviations

ACL: Anterior cruciate ligament
APD: Anterior-posterior diameter
ICCs: Intraclass correlation coefficients
MRI: Magnetic resonance imaging
PCL: Posterior cruciate ligament
PTS: Posterior tibial ‘shelf’
